# *Aedes aegypti*, *Ae. albopictus* and *Culex quinquefasciatus* Adults Found Coexisting in Urban and Semiurban Dwellings of Southern Chiapas, Mexico

**DOI:** 10.3390/insects14060565

**Published:** 2023-06-17

**Authors:** Alma D. Lopez-Solis, Francisco Solis-Santoyo, Karla Saavedra-Rodriguez, Daniel Sanchez-Guillen, Alfredo Castillo-Vera, Rebeca Gonzalez-Gomez, Americo D. Rodriguez, Patricia Penilla-Navarro

**Affiliations:** 1 Centro Regional de Investigación en Salud Pública, Instituto Nacional de Salud Pública, Cuarta Norte y 19 Calle Poniente S/N Colonia Centro, Tapachula 30700, Mexico; adlopez@insp.mx (A.D.L.-S.); americo.rodriguez@hotmail.com (A.D.R.); 2 El Colegio de la Frontera Sur, Unidad Tapachula. Carretera Antiguo Aeropuerto Km. 2.5, Centro, Tapachula Chiapas 30700, Mexico; dsanchez@ecosur.mx (D.S.-G.); acastill@ecosur.mx (A.C.-V.); rgonzalez@ecosur.mx (R.G.-G.); 3 Arthropod-Borne and Infectious Diseases Laboratory, Department of Microbiology, Immunology, and Pathology, College of Veterinary Medicine and Biomedical Sciences, Colorado State University, 1692 Campus Delivery, Fort Collins, CO 80523-1692, USA; karla.saavedra_rodriguez@colostate.edu; 4 Investigadora por México, Consejo Nacional de Humanidades, Ciencias y Tecnologías, Av. Insurgentes Sur 1582, Benito Juárez 03940, Mexico

**Keywords:** disease vectors, coexistence, vectors, adults mosquitoes, indoors, outdoors

## Abstract

**Simple Summary:**

*Aedes aegypti*, *Ae albopictus,* and *Culex quinquefasciatus*, three mosquito species of medical importance, were found coexisting in residential neighborhoods of urban and semiurban areas. *Aedes aegypti* was mostly present indoor houses compared to *Ae. albopictus* and *Cx. quinquefasciatus*. On the contrary, in cemeteries of the urban area, *Ae. aegypti* was found in lower densities compared to *Ae. albopictus* and *Cx. Quinquefasciatus*, which were the most abundant. The identification of these species and the knowledge of their distribution are essential for entomological surveillance in the prevention of outbreaks of vector-borne diseases.

**Abstract:**

Tapachula, Mexico, a tropical city, is an endemic area for dengue, in addition to several outbreaks in the last decade with chikungunya and zika. As part of the migratory corridor from Central to North America and the risks of scattered infectious diseases that this implies, the identification and distribution of potential disease vectors in and around residential areas are essential in terms of entomological surveillance for the prevention of disease outbreaks. The identification of mosquito species of medical importance coexisting in houses and cemeteries in Tapachula and two semiurban sites in southern Chiapas was investigated. Adult mosquitoes were collected from May to December 2018, resting inside and outside houses and in the tombstones and fallen tree leaves in cemeteries. A total of 10,883 mosquitoes belonging to three vector species were collected across 20 sites; 6738 were from neighborhood houses, of which 55.4% were *Culex quinquefasciatus*, 41.6% *Aedes aegypti*, and 2.9% *Ae. albopictus*. *Aedes aegypti* was the most common mosquito resting inside houses (56.7%), while *Ae. albopictus* and *Cx. quinquefasciatus* were mostly found resting outside houses (75.7%). In the cemeteries, *Cx. quinquefasciatus* (60.8%) and *Ae. albopictus* (37.3%) were the most abundant, while *Ae. aegypti* (1.9%) was the least abundant. This is the first report to identify adults of three major disease vector species coexisting in the domestic environment of urban and semiurban sites and *Ae. albopictus* adult resting inside of urban houses in Mexico. It would be opportune to consider comprehensive strategies that can be applied in this region to control the three species at the same time and avoid outbreaks of the diseases they transmit.

## 1. Introduction

The last century has witnessed a wave of severe infectious disease outbreaks [1], which occurred in urban, peri-urban, and rural communities. It is believed that they are predominantly found among communities that have poor living conditions, particularly a lack of access to adequate housing, clean water, and sanitation. There are no vaccines for many vectors-borne diseases, and drug resistance is a growing threat. Therefore, vector control plays a vital role; so far, it is the principal way to prevent disease outbreaks [2]. Mosquitoes are vectors known as spreaders of viruses, bacteria, protozoa, and nematodes [3]. *Aedes aegypti* is the main species responsible for the transmission of dengue in the world. It is believed that its introduction to the Americas was by European travelers, causing the first epidemic in Mexico, yellow fever, which was widely distributed throughout the country [4]. The dengue epidemic in Mexico at the end of the 1970s was also due to *Ae. aegypti*.

The Chiapas State, particularly Tapachula, was the gateway for dengue [5]. To date, Tapachula is an endemic area for dengue (DENGV), which also had outbreaks of chikungunya (CHIKV) (2014) and Zika virus (ZIKV) (2015) infection [6,7], with *Ae. aegypti* as the vector involved. *Aedes aegypti* is widely distributed and adapted to domestic environments, it is characterized by adults with diurnal feeding characteristics that generally feed and rest indoors [8]. Meanwhile *Ae. albopictus* is becoming a potential vector of dengue in Mexico because it is now spreading in hyperendemic areas for dengue, where the four DENGV serotypes circulate [9]. It was found in Tapachula in 2002 [10], where the four DENGV serotypes also circulate [11]. However, only wild male mosquitoes have been found to be infected with DENGV serotypes 2 and 3 in the north of the country, during an outbreak in the city of Reynosa in 1995 [12], and transovarial transmission was reported to be occurring naturally during the summer of 2010 in a suburban region near Monterrey, Northeast Mexico [13]. This species has also been implicated as the main vector in chikungunya outbreaks in Italy [14] and in Zika outbreaks in South America [15]. The first chikungunya outbreak in Italy that occurred in 2007 was with a virus strain with the E1:A26V mutation, while in the 2017 outbreak, the virus did not present with a mutation, which suggests that the two viral strains infect, spread, and transmit in a similar way [16]. Mosquito populations from the Americas stood out in terms of their ability to transmit three CHIKV genotypes, with transmission rates of up to 96%, suggesting a high risk of establishment and spread [17]. However, given its limited transmission compared to that of *Ae. aegypti* due to its low vector competence [18], in Mexico, it is considered to be a potential disease vector since it was recently found to be infected with ZIKV but in the absence of confirmed symptomatic human cases [19]. It is believed that *Ae. albopictus* prefers environments with more vegetation, but studies have demonstrated the presence of larvae under artificial breeding conditions occupied by *Ae. aegypti* [20]. Therefore, it is considered to be a species with ecological plasticity, which allows it to adapt to new environments [21]. 

*Culex quinquefasciatus* is another common mosquito species found in Mexico, distributed throughout the country and throughout the year. This species, together with *Ae. Aegypti*, represents a risk of contact with human populations in urban and rural environments [22]. Its main characteristic is the great variety of natural and artificial habitats with abundant organic matter that the larval stages occupy [8]. Furthermore, *Cx. Quinquefasciatus* is the main vector of lymphatic filariasis [23], St. Louis encephalitis virus (SLEV), and West Nile virus in the southern United States. In Mexico, the states of Coahuila, Yucatán, and Chihuahua reported West Nile Virus, with *Cx. quinquefasciatus* being the main vector [24,25,26]. This species has been found to be refractory to the infection, dissemination, and transmission of ZIKV in Guadalajara and Mexico City [27].

Given the importance of *Ae. aegypti* as the main vector of infectious diseases throughout Mexico, many of its behavioral characteristics are already known, such as its preference for resting indoors [28,29]; therefore, vector control programs currently focus on its ethological dynamics in order to coordinate activities for its control. However, *Ae. albopictus* and *Cx. quinquefasciatus* are also potential disease vectors; therefore, they should not be left unattended. Entomological studies in the domestic environments of these two species are limited, and generally, those of population abundance are carried out using ovitrap and larval collection methods, from which, at the same time, information associated with their reproduction habits is also obtained. This work was based on collecting adult mosquitoes that rest inside and outside of houses, which made it possible to evaluate both the interactions between the mosquito species/humans and the coexistence between the mosquito species themselves. In addition to being an endemic region for dengue and with outbreaks of CHIKV and ZIKV transmission, Tapachula is part of the migratory corridor of human movements to the north of the American continent. Its location, environmental and endemic conditions, and the surroundings make it important to evaluate the identification and distribution of the disease vector species. To find out if different species of disease vectors coexist in the residences of Tapachula and the surroundings and in two semiurban sites, adult mosquitoes were collected from outside and inside of houses across 20 sites, including two cemeteries, where identification, abundance, and resting behavior were recorded. 

This information will be of interest in terms of the entomological surveillance of vector-borne diseases, focused on optimizing the methods that are used to control the adults of the vector species that coexist in houses of both urban and semiurban areas to avoid outbreaks of the diseases they transmit.

## 2. Materials and Methods

### 2.1. Study Area

The study was carried out in Tapachula, Chiapas (14°54′28″ N and 92°15′28″ W), an urban site with 305,766 inhabitants with a territorial extension of 904 km^2^ and an altitude of 170 m above sea level. It is located 26 km from Guatemala’s border to the north of Mexico. It has a warm and humid tropical climate with rain from May to November, with a mean annual temperature of 33 °C and a minimum of 25 °C, and there was normal temperature and rainfall variation during 2018. The rivers of Texcuyuapan and Coatán, forming the Coatancito Creek, cross the city in a North–South direction. Since 1970, dengue has been endemic in the city [5], with recently important CHIKV and ZIKV outbreaks. The coordinates for the semiurban sites, where the mosquito collections were also undertaken, Puerto Madero and Mazatán, are located 27.8 km to the southwest and 26.6 km to the west from Tapachula, respectively.

### 2.2. Mosquito Collection

**Figure 1:**
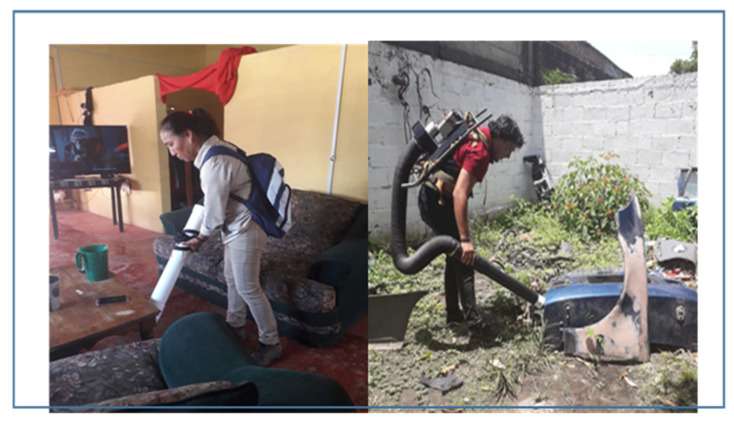
Collections of adult mosquitoes inside and outside houses and in cemeteries using Insecta Zooka Aspirators (**left**) and Backpack Aspirators model 1412 (**right**) in Tapachula, Puerto Madero, and Mazatán, Chiapas, Mexico.

Mosquitoes were collected across 20 sites, wherein 16 neighborhoods and two cemeteries (18 urban sites) were searched for adult mosquitoes in Tapachula and in 2 neighborhoods of the semiurban sites. The mosquito collections were conducted from May to December 2018 from a total of 350 houses from 9:00 a.m. to 2:00 p.m. The number of houses that were sampled per site (Table 1) depended on the number of houses located in the central block of each site and houses in the adjacent blocks. Furthermore, the owner’s permission was obtained before searching for mosquitoes inside and outside of the houses. In each block, the collection began in the first house located in the northernmost corner. This procedure was always performed in a clockwise direction. The number of houses collected at each site is shown in Table 1. Adult mosquitoes were collected with backpack aspirators (Backpack Aspirator model 1412) and portable compact aspirators (Insecta Zooka) (Figure 1). In the houses, mosquitoes were searched for around furniture, curtains, and coat racks, in dark and humid places, and on wall surfaces, where they usually rest at a height no higher than 1.5 m when inside houses. The outdoor mosquito collections were carried out by searching plants, animal houses, and areas protected from the sun’s rays. The average vacuuming time per house was 10 min. 

The caught mosquitoes were labeled, indicating the study site, house number, vacuuming area (indoors or outdoors), and date of collection. All of the collected specimens were transferred to the laboratory of the Centro Regional de Investigación en Salud Pública (CRISP) for species and sex identification. Species identification was carried out with a stereoscopic microscope, using the identification keys of Rueda [30] for *Ae. aegypti* and *Ae. albopictus* and the keys of Darsie RF et al. [31] for *Cx. quinquefasciatus* According to the classic taxonomic identification and morphological characteristics of each species, no elements were found that indicate cryptic species; however, this possibility has not been ruled out; since it was not included in the objectives, no test was performed in relation to this, but it may be suggested for further study. Mosquito collections were carried out once a month in each block. Six sites were collected only twice (Table 1), but the rest of the sites were collected three times, which depended on the abundance of mosquitos at each site. For the aspirations to be homogeneous across all of the study sites, the same 6–8 technicians carried out the vacuuming activities.

The two cemeteries visited to perform the mosquito collections are public; the Municipal cemetery is near the center of Tapachula, while the Jardin cemetery is located to the east of Tapachula, and both are surrounded by houses. They were visited three times, and collections were undertaken on the surroundings of the graves, vases, plantings, tree trunks, and fallen leaves. The sampling time for each visit to the cemeteries was 30 min, with six people performing the collections with four Insecta Zooka Aspirators and two Backpack Aspirators (model 1412). All of the sites were geo-referenced using a positioning system receiver (GPS/Garmin) (Table 1).

### 2.3. Statistical Analyses

The mean ± standard deviation of mosquito abundance, the three mosquito species, and those captured indoors and outdoors were calculated and compared using a one-factor ANOVA test and Dunnett’s post hoc test or a *t*-test for independent samples to detect the differences between them, with a significance of 95%, using IBM SPSS Statistics v.26. To find out the differences in the abundance between the indoor and outdoor collections, R Studio statistical software was used for statistical computing. Non-parametric 95% bootstrap CIs were calculated by taking 1000 bootstrap samples with a replacement for a month within site for site-wise statistics. The means were calculated from each bootstrap sample, and 2.5% and 97.5% quantiles of the sorted distribution were found. Finally, the following entomological indices were calculated for the three species: positive house index (PHI) and mosquito density/house (F/H) [32], which also resulted in the distribution of the three species across the 20 collection sites. Spearman’s correlation coefficient test was also applied to find out if the abundance of mosquitoes in the houses of the neighborhoods depended on the environmental temperature of the city.

## 3. Results

A total of 10,883 mosquitoes were collected from May to December 2018 across all 20 collection sites, of which 6255 (57.5%) were *Cx. quinquefasciatus* (mean ± standard deviation: (30.66 ± 87.18), 2888 (26.5%) were *Ae. aegypti* (14.16 ± 16.50), and 1740 (16%) were *Ae. albopictus* (8.53 ± 36.32). Statistical differences were obtained between the abundance of both species of *Aedes* (*p* ˂ 0.025) vs. *Cx. quinquefasciatus*, but no statistical differences were found between *Aedes* species across the 20 sites.

Mostly, the mosquitoes were collected from neighborhood houses (6738, sites = 18), of which 3736 (55.4%) were *Cx. quinquefasciatus* (19.46 ± 50.74), 2807 (41.6%) were *Ae. aegypti* (14.62 ± 16.77), and only 195 (2.9%) were *Ae. albopictus* (1.02 ± 2.40). Statistical differences were only obtained between the abundance of *Ae. albopictus* vs. *Ae. aegypti* (*p* < 0.0001) and vs. *Cx. quinquefasciatus* (*p* < 0.0001), but there were no statistical differences between *Ae. aegypti* vs. *Cx. quinquefasciatus*. No statistical differences were obtained in terms of the total number of mosquitoes collected between the urban sites (5401, *n* = 84 collections, 64.30 ± 104.95) and the semiurban sites (1337, *n* = 12, 111.42 ± 93.86), either in the *Ae. aegypti* collected between the urban sites (2451, *n* = 84, 29.18 ± 32.05) or the semiurban sites (356, *n* = 12, 29.67 ± 25.99).

A total of 3609 (53.6%) mosquitoes were collected indoors (200.50 ± 157.92), and 3129 (46.4%) mosquitoes were collected outdoors (173.83 ± 230.45), but no statistical differences were found in terms of mosquito abundance between outdoors and indoors of houses of the 18 sites. Of which 2046 (56.7%) were *Ae. aegypti* (21.31 ± 19.11), 1511 (41.9%) were *Cx. quinquefasciatus* (15.74 ± 36.23), and only 52 (1.4%) were *Ae. albopictus* (0.54 ± 1.23) from indoors. Statistical differences were obtained between the abundance of *Ae. albopictus* vs. *Ae. aegypti* (*p* < 0.0001) and vs. *Cx. quinquefasciatus* (*p* < 0.0001), but there were no differences between *Ae. aegypti* vs. *Cx. quinquefasciatus*. Outdoors, a total of 3129 were collected, of which 761 (24.3%) were *Ae. aegypti*, (7.93 ± 10.50), 2225 (71.1%) were *Cx. quinquefasciatus* (23.18 ± 61.93), and only 143 (4.6%) were *Ae. albopictus* (1.49 ± 3.01). Statistical differences were only obtained between the abundance of both *Aedes* species (*p* < 0.0001) across the 18 sites collected. The number of mosquitoes for the three surveys and the ratios of males and females by indoor and outdoor collections for the three species are shown in Table 2. 

*Aedes aegypti* males and females were more often collected indoors, while both males and females of *Ae. albopictus* and *Cx. quinquefasciatus* were collected outdoors more often. The male ratios were always the highest for all three mosquito species, both indoors and outdoors of the houses. In the Spearman correlation coefficient test, a negative correlation between the number of mosquitoes collected per site (*n* = 18 sites) inside the houses and the maximum temperature recorded in the city for the days of the collections was only found for the first of the two or three collections made in each site (*p* < 0.05). The lower the temperature, the greater the abundance of mosquitoes inside the houses.

**Figure 2:**
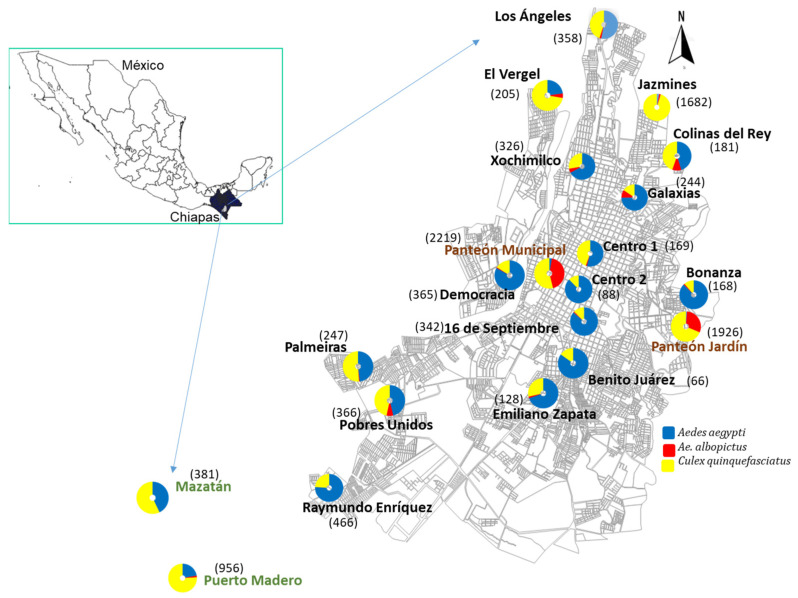
Spatial distribution of *Aedes aegypti*, *Ae. Albopictus*, and *Culex quinquefasciatus* across 16 sites of Tapachula and at the two semiurban sites: Mazatán and Puerto Madero. In addition, two urban cemeteries were included in the study in Tapachula. The total of mosquitoes collected per each of the 20 sites is shown in brackets. The site names in black represent urban areas, in green semiurban areas, and in brown, cemeteries.

The infestation house index or PHI per site for each species is shown in Table 3. *Aedes aegypti* had the highest PHI, with Centro 1, Democracia, Bonanza, 16 de Septiembre, Emiliano Zapata, and Palmeiras with 100%. Jazmines had the lowest PHI (45%). Interestingly, in the same sites with the lowest *Ae. aegypti* infestation, *Cx. quinquefasciatus* was found to have the highest infestation indices. In general, *Ae. albopictus* was recorded as having the lowest infestation in dwellings, ranging from 1% to 10%. The spatial distribution of the mosquito collection sites is shown in Figure 2. *Aedes aegypti* is present in most of the sites, with less abundance in Jazmines and in both cemeteries. The F/H index also situates *Ae. Aegypti* as being the most abundant most of the time (Table 3), with a minimum of five and a maximum of eighteen, followed by *Cx. Quinquefasciatus*, from 2 to 80, and *Ae. albopictus* as the least abundant, from 0 to 8. *Culex quinquefasciatus* had a maximum F/H index of 80 because, in one of the houses surveyed in Jazmines, 95% of the mosquitoes collected belonged to this species, with 686 collected outdoors (368 males and 318 females), and 75 collected indoors (48 males and 27 females). Based on 1000 samples, the Bootstrap analysis showed *Ae. aegypti* as the most abundant inside houses, in 10 out the 18 sites (Table 3). While *Cx. quinquefasciatus* and *Ae. albopictus* only showed a greater abundance indoors than *Ae. aegypti* in two (Xochimilco y Galaxias) and three sites (Vergel, Xochimilco, and Raymundo Enriquez), respectively. Xochimilco was the most infested site, where the three species had the highest abundance inside houses.

**Figure 3:**
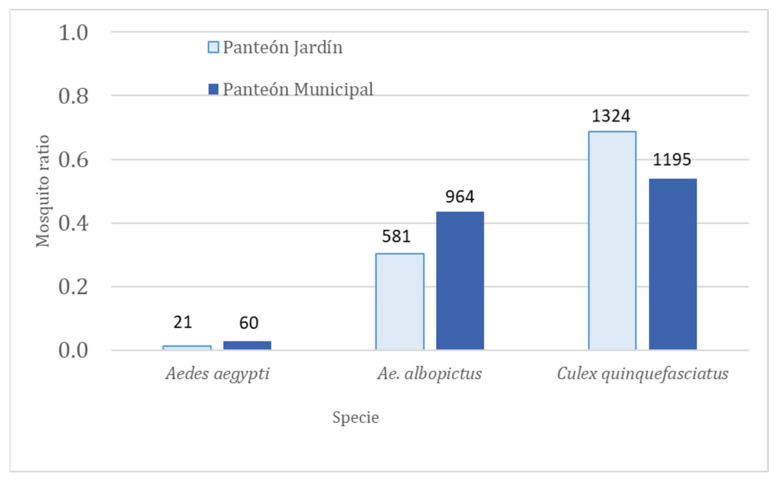
The ratio of mosquitoes collected from June to December 2018 in two cemeteries in Tapachula, Chiapas, México. The number on the bar indicates the number of mosquitoes collected. Mosquito ratio = the number of mosquitoes of each species collected ÷ the total number of mosquitoes collected in each cemetery.

In the cemeteries, a total of 4145 mosquitoes belonging to the three species were collected, *Cx. quinquefasciatus* was the most abundant with 2519 (61%) (839.67 ± 1088.21), followed by *Ae. albopictus* with 1545 (37%) (515.0 0 ± 235.15), and the least abundant was *Ae. Aegypti* with 81 (2%) (27.00 ± 15.71) (Figure 3). However, no statistical differences in terms of abundance were obtained between the three species of mosquitoes in the three collections carried out. The ratio of females for *Ae. Aegypti* was 0.4 and 0.2 for Panteón Jardín and Panteón Municipal, respectively. For *Ae. Albopictus*, the ratio of females was 0.9 and 0.8, while for *Cx. Quinquefasciatus*, the ratio was 0.8 for both cemeteries (Table 4).

## 4. Discussion

In this study, three disease vector mosquito species were identified as coexisting in houses and their surroundings, and their abundance and resting behavior are reported for an urban (Tapachula) and two semiurban sites (Mazatán and Puerto Madero) in southern Chiapas, Mexico. Furthermore, it is the first report of *Ae. albopictus* adults found resting inside of the dwellings. It is well known that arboviruses have increased due to the disturbances of ecosystems caused by commercial globalization and social migrations; such anthropic changes affect natural mosquito populations changing their ecological habits and consequently influencing the dynamics of pathogens within the habitat of the human environment [33]. A wide distribution of *Ae. aegypti* and *Cx. quinquefasciatus* mosquito populations were found in Tapachula and in both semiurban sites, Puerto Madero and Mazatán. While *Ae. albopictus* was much less abundant and was not always found at the collected sites, as was seen at four of the eighteen sites when collected from houses.

Higher proportions of *Ae. aegypti* females collected from inside houses were reported by Dzul-Manzanilla et al. [29] in Guerrero, Mexico (99%), and by Chadee DD [34] in Trinidad (81.9%), than those reported in this study (73.5%), proportions that might differ depending on when the mosquito searches were conducted in each site since it has been reported that abundance differs between the rainy and dry seasons [35]. Nevertheless, those results confirm the endophilic behavior that *Ae. aegypti* possesses and reaffirms its importance in terms of its the ability to transmit pathogens via the predisposition of human feeding indoors [36]. *Aedes aegypti* has been the main vector of dengue since the outbreak of 1997 in the sites surveyed for this study, and it is a species predominantly found in urban, semiurban, and rural areas. Additionally, it was incriminated in the transmission of the emerging diseases of CHIK [6] and ZIKV [7] in Tapachula.

*Aedes albopictus* has been found in semiurban areas in Merida, Mexico, where up to eight mosquitoes were collected inside houses via human bait collections; another study from Mexico City reported collecting *Ae. albopictus* in ovitraps [8], but it is not unknown to find this species in vacant lots with extensive vegetation [37]. In 2003, Casas-Martínez M et al. [10] reported for the first time the presence of this species on the outskirts of Tapachula. All of these findings indicate the coexistence of both *Ae. aegypti* and *Ae. albopictus* species. Despite the fact that *Ae. albopictus* has been considered to be less competent than *Ae. Aegypti* [18], strains from the Americas have shown high rates of infection and transmission under experimental conditions; in some cases, the rates for *Ae. Albopictus* are even higher than those of *Ae. Aegypti* [38]. However, in Mexico, it is still considered to be a potential disease vector since, despite its presence in almost the entire national territory, only a few male mosquitoes infected with DENGV with serotypes 2 and 3 in Tamaulipas, and transovarial infection by a few females reared to adults from eggs collected in ovitraps, both from northeast Mexico, have been reported as occurring naturally [12,13].

However, this implies that if the virus is efficiently transmitted to its progeny, the virus can persist during inter-epidemic periods [39]. There is evidence of the presence of *Ae. albopictus* in semiurban and rural areas [40], including evidence of this species using the same oviposition sites as *Ae. aegypti* [41]. Nevertheless, this study is the first to report the presence of adult *Ae. albopictus* mosquitoes inside urban houses. This information is important as evidence for the surveillance and control of this species in urban and semiurban areas since it is a competent species for at least 22 arboviruses [42].

*Culex quinquefasciatus* is widely distributed in Mexico and is found throughout the whole year [26] in a wide variety of natural and artificial environments with abundant organic matter [8]. It is the main vector of SLEV and is related to the West Nile virus [43]. Additionally, it was found to be refractory to the infection, dissemination, and transmission of the ZIKV [27]. We report the presence of this mosquito at all study sites with variable infestations. The abundance of *Cx. quinquefasciatus* was higher in urban fringe areas (Figure 3), which are considered to be sites that lack public services, housing with less infrastructure, and poor welfare conditions. An example is Jazmines, which is located at the limits of Tapachula, which is characterized by regular vegetation, it does not have paved streets and the socioeconomic level is low, wherein a greater abundance of *Cx. quinquefasciatus* was found in 7 of the 22 houses, with an average of 50 to 170 mosquitoes per house. While in Puerto Madero, a semiurban area, with no paved streets except for access roads, this species was found in 20 of the 24 sampled houses, with an average of 20 to 83 mosquitoes per house. This suggests that the abundance of this species may also be influenced by housing conditions. Therefore, the proliferation of this mosquito species is favored with possible breeding sites with abundant organic matter. On the contrary, *Ae. aegypti* was recorded as having a lower abundance in these areas because it is a species that reproduces in natural and artificial containers that contain clear and clean water [44].

*Aedes albopictus* was the least abundant species, and it is assumed that the time in which the collections were undertaken influenced these results since *Ae. albopictus* from this region is susceptible to insecticides [45,46], and spray activity by the local control program was active during 2018, which could keep the adult populations at low levels. However, a pattern of preference for resting in the peri-domestic area was observed. Previous studies carried out on collections of larvae have reported the presence of this species in outdoor domestic areas [47]. Vector infestation in new areas can be a risk factor for possible infections [48]. In addition to the abundance of mosquitoes, it generates a negative impact on the quality of life in the human environment. 

Our study enhances the importance of reporting the findings of anthropophilic vector species, mainly in endemic areas of diseases of medical importance. It has been speculated that competition from the vector *Ae. albopictus* is positively associated with colonization time. For this reason, it also applies to the importance of monitoring it since it can also serve as a binding vector that transports viruses to domestic environments and, therefore, increases the risk to humans [1,18].

Studies carried out focusing on urban species in urban green spaces found a relationship between area and species richness, suggesting that green areas tend to reduce the risk of the extinction of specialized species [49]. On the other hand, reports on the abundance of *Ae. aegypti* in urban areas without green spaces are statistically significant, indicating greater abundance [50]; this species is found mostly in residential areas. Like our results, where we reported *Ae. aegypti* as the least dominant species in the sampled cemeteries; on the contrary, *Ae. albopictus* and *Cx. quinquefasciatus* recorded higher numbers of mosquitoes. A recent study concluded that there is no significant evidence to validate the concern that green spaces increase exposure to and the risk of mosquito-borne diseases [44].

## 5. Conclusions

The presence of *Ae. aegypti* across all of the study sites confirms its wide distribution in urban and semiurban areas, being the species with greater contact with humans due to their preference for the interiors of houses. The distribution and abundance of vector species are important factors that favor arboviral diseases; therefore, the coexistence of *Ae. Aegypti*, *Ae. Albopictus*, and *Cx. quinquefasciatus* in domestic settings make it a high-risk area for vector-borne disease outbreaks. This study is the first study in Mexico to report the presence of adult *Ae. albopictus* mosquitoes resting inside the houses of an urban city.

## Figures and Tables

**Table 1:** The coordinates of the 20 sites and the number of houses per site where adult mosquitoes were collected from May to December 2018 in Tapachula, Puerto Madero, and Mazatán, Chiapas, Mexico.

No	Sites	Coordinates			
		Latitude	Longitude	Houses Collected	Months of Mosquito Collections
1	El Vergel	14°56′21.2″	92°15′52.4″	18	May, June, December
2	Los Ángeles	14°56′42.3″	92°15′21.2″	24	May, July, November
3	Jazmines	14°53′32.2″	92°17′19.4″	22	May, June, November
4	Xochimilco	14°55′48.9″	92°15′37.8″	22	May, June, November
5	Colinas del Rey	14°55′50.9″	92°14′50.2″	17	May, June, November
6	Galaxias	14°55′11.2″	92°15″06,5″	13	May, June, November
7	Centro 1	14°54′22.7″	92°15‘32.8″	15	June, July, November
8	Democracia	14°54′23.7″	92°16′33.5″	28	June, July, November
9	Panteón Municipal	14°54′15.3″	92°16′13.1″	-	June, August, December
10	Centro 2	14°54′ 8.5″	92°15′47.3″	17	June, July, November
11	Bonanza	14°54′02.8″	92°14′31.7″	28	May, July, November
12	Panteón Jardín	14°53′41.7″	92°14′56.6″	-	June, August, November
13	16 de Septiembre	14°53′44.0″	92°15′42.1″	16	May, July
14	Benito Juárez	14°53′21.8″	92°16′04.1″	7	May, July
15	Emiliano Zapata	14°53′02.1″	92°16′14.2″	16	May, July
16	Raymundo Enríquez	14°52′01.4″	92°18′48.8″	25	May, June
17	Pobres Unidos	4°53′14.0″	92°17′6.1″	25	June, July
18	Palmeiras	14°53′22.1″	92°18′06.4″	13	June, July
19	Puerto Madero	14°43′21.7″	92°25′38.7″	24	June, August, November
20	Mazatán	14°52′3.16″	92°26′59.88″	20	Jule, August, November

**Table 2:** The number of male and female mosquitoes collected by species indoor and outdoor of 350 houses from May to December 2018. The ratios of males and females are in the parenthesis.

House Area	Collection Time	*Aedes aegypti* (2807)		*Ae. albopictus* (195)		*Culex quinquefasciatus* (3736)	
		Male	Female	Male	Female	Male	Female
Indoors (3609)	1	488 (1.19)	407 (0.83)	17 (1.30)	13 (0.76)	437 (1.12)	389 (0.89)
	2	492 (1.17)	417 (0.84)	7 (1.00)	7 (1.00)	189 (1.35)	139 (0.73)
	3	164 (2.10)	78 (0.47)	5 (1.66)	3 (0.60)	230 (1.81)	127 (0.55)
	Total	1144 (1.26)	902 (0.78)	29 (1.26)	23 (0.79)	856 (1.30)	655 (0.76)
Outdoors (3129)	1	202 (1.43)	141 (0.69)	51 (3.40)	15 (0.29)	418 (1.24)	336 (0.80)
	2	225 (1.71)	131 (0.58)	29 (0.96)	30 (1.03)	274 (1.21)	225 (0.82)
	3	38 (1.58)	24 (0.63)	10 (1.25)	8 (0.80)	555 (1.33)	417 (0.75)
	Total	465 (1.57)	296 (0.63)	90 (1.69)	53 (0.58)	1247 (1.27)	978 (0.78)

Ratios of males = the number of male mosquitoes ÷ number of female mosquitoes. Ratios of females = the number of female mosquitoes ÷ number of male mosquitoes.

**Table 3:** Positive house index, mosquito density/house, and Bootstrap analysis abundance of mosquitoes inside of 350 houses collected in Tapachula, Puerto Madero, and Mazatán, Chiapas, Mexico.

	Index Positive House			Mosquito Density/House			Bootstrap (95% CI)		
Site	*Aedes aegypti*	*Ae. albopictus*	*Culex quinquefasciatus*	*Ae. aegypti*	*Ae. albopictus*	*Cx. quinquefasciatus*	*Ae. aegypti*	*Ae. albopictus*	*Cx. quinquefasciatus*
Vergel	78	22	94	5	2	8	*		*
Los Ángeles	83	17	71	10	2	9	*		
Jazmines	45	18	91	5	8	80			
Xochimilco	95	36	45	11	3	8	*	*	*
Colinas del Rey	76	35	88	7	3	5	*		
Galaxias	85	62	69	16	3	4	*	*	
Centro 1	100	87	60	5	1	10	*		
Democracia	100	14	46	11	1	4	*		
Centro 2	82	0	29	6	0	2			
Bonanza	100	7	50	5	1	2	*		
16 de Septiembre	100	19	50	18	1	6			
Benito Juárez	86	0	29	9	0	5			
Emiliano Zapata	100	19	56	5	3	4	*		
Raymundo Enríquez	92	0	60	15	0	8	*		*
Pobres Unidos	88	24	56	7	7	12			
Palmeiras	100	15	23	9	1	41			
Puerto Madero	92	33	79	10	2	38			
Mazatán	90	0	85	8	0	14			

* Significance of the abundance indoors house vs. outdoors is because their confidence intervals do not overlap.

**Table 4:** The number of males and females mosquitoes collected by species in the cemeteries from May to December 2018. The ratios of males and females are in the parenthesis.

		*Aedes aegypti* (81)		*Ae. albopictus* (1545)		*Culex quinquefasciatus* (2519)	
Site	Collections Time	Male	Female	Male	Female	Male	Female
Panteón Jardín	1	2 (0.66)	3 (1.50)	97 (0.98)	98 (1.01)	672 (1.23)	543 (0.80)
(1926)	2	7 (7.00)	1(0.14)	91 (1.04)	87 (0.95)	66 (2.53)	26 (0.39)
	3	6 (3.00)	2 (0.33)	122 (1.41)	86 (0.70)	13 (3.25)	4 (0.30)
	Total	15 (2.50)	6 (0.40)	310 (1.14)	271 (0.87)	751 (1.31)	573 (0.76)
Panteón Municipal	1	19 (3.16)	6 (0.31)	188 (0.89)	209 ((1.11)	459 (1.08)	422 (0.91)
(2219)	2	29 (7.25)	4 (0.13)	321 (1.58)	203 (0.63)	47 (0.90)	52 (1.10)
	3	0 (0.00)	2 (2.00)	22 (1.00)	21 (0.95)	140 (1.86)	75 (0.53)
	Total	48 (4.00)	12(0.25)	531(1.22)	433 (0.81)	646 (1.17)	549 (0.84)

## Data Availability

No new data were created or analyzed in this study. Data sharing is not applicable to this article.
